# In vivo human brain expression of histone deacetylases in bipolar disorder

**DOI:** 10.1038/s41398-020-00911-5

**Published:** 2020-07-08

**Authors:** Chieh-En J. Tseng, Tonya M. Gilbert, Mary C. Catanese, Baileigh G. Hightower, Amy T. Peters, Anjali J. Parmar, Minhae Kim, Changning Wang, Joshua L. Roffman, Hannah E. Brown, Roy H. Perlis, Nicole R. Zürcher, Jacob M. Hooker

**Affiliations:** 1 Athinoula A. Martinos Center for Biomedical Imaging, Department of Radiology, Massachusetts General Hospital, Harvard Medical School, Charlestown, MA 02129 USA; 2 Department of Psychiatry, Massachusetts General Hospital, Harvard Medical School, Boston, MA 02114 USA; 3 Department of Psychiatry, Boston University School of Medicine, Boston, MA 02118 USA; 4 Center for Genomic Medicine, Massachusetts General Hospital, Harvard Medical School, Boston, MA 02114 USA

**Keywords:** Epigenetics and behaviour, Bipolar disorder, Molecular neuroscience

## Abstract

The etiology of bipolar disorder (BD) is unknown and the neurobiological underpinnings are not fully understood. Both genetic and environmental factors contribute to the risk of BD, which may be linked through epigenetic mechanisms, including those regulated by histone deacetylase (HDAC) enzymes. This study measures in vivo HDAC expression in individuals with BD for the first time using the HDAC-specific radiotracer [^11^C]Martinostat. Eleven participants with BD and 11 age- and sex-matched control participants (CON) completed a simultaneous magnetic resonance – positron emission tomography (MR-PET) scan with [^11^C]Martinostat. Lower [^11^C]Martinostat uptake was found in the right amygdala of BD compared to CON. We assessed uptake in the dorsolateral prefrontal cortex (DLPFC) to compare previous findings of lower uptake in the DLPFC in schizophrenia and found no group differences in BD. Exploratory whole-brain voxelwise analysis showed lower [^11^C]Martinostat uptake in the bilateral thalamus, orbitofrontal cortex, right hippocampus, and right amygdala in BD compared to CON. Furthermore, regional [^11^C]Martinostat uptake was associated with emotion regulation in BD in fronto-limbic areas, which aligns with findings from previous structural, functional, and molecular neuroimaging studies in BD. Regional [^11^C]Martinostat uptake was associated with attention in BD in fronto-parietal and temporal regions. These findings indicate a potential role of HDACs in BD pathophysiology. In particular, HDAC expression levels may modulate attention and emotion regulation, which represent two core clinical features of BD.

## Introduction

The etiology and underlying pathology of bipolar disorder (BD) are poorly understood, and their elucidation is complicated by misdiagnosis due to overlapping symptoms with other neuropsychiatric disorders including schizophrenia, unipolar depression, and impulse control^1^. Polygenic risk has explained some of the heritability of BD, which is estimated at ~60–80%^2–4^, and environmental factors including childhood trauma and life events contribute additional risk^5^. The connection between these two variables—genetic and environmental risk—has been difficult to assess but may be explained at a molecular level by epigenetic mechanisms. Moreover, dysregulation of gene transcription in animal models of neuropsychiatric disorders is well-established and in part can be attributed to epigenetic enzymes, including histone deacetylases (HDACs)^6,7^.

Genetic association and clinical pharmacology have been used to identify a potential role for HDACs in BD. For example, a recent systematic analysis of genome-wide association study (GWAS) data incorporating gene pathway analysis, found that *HDAC2* may be linked to increased genetic risk for BD through its involvement in the development of the amygdala, nucleus accumbens and hippocampus^8^. Moreover, pharmacological treatments for BD act on HDACs. Specifically, valproic acid (VPA) is an HDAC inhibitor^9^, lithium downregulates HDAC1^10^, and lamotrigine increases histone acetylation levels in vitro^11^. Additionally, hyper-activity was reduced by HDAC inhibition in preclinical models of mania^12,13^. Collectively, these observations beg the question of whether HDACs may represent a direct mechanistic link to BD.

Brain pathology has implicated a potential association between HDACs and BD in specific brain regions. For example, in the dorsolateral prefrontal cortex (DLPFC) and caudate, no differences were detected in *HDAC1* or *HDAC2* mRNA transcript levels between BD donor samples compared to controls^14^, while in the hippocampus SO-CA2/3 region, *HDAC1* mRNA transcript levels were ~3-fold lower in BD donor samples compared to controls^15^. However, pathology studies are fraught with classical challenges in interpreting results from postmortem tissue, including the analysis of a limited number of brain regions across studies with varying methodology.

Evidence from rodent studies also suggest involvement of HDACs in relation to BD-associated behavior or treatment. For example, anti-manic effects of HDAC inhibition are suggested to be related to the amygdala, striatum, and prefrontal cortex, but not the hippocampus in rats^12^. Also, treatment of lithium or the HDAC inhibitor, sodium butyrate, increased the levels of acetylation on histone H3, an indirect measure of altered HDAC levels and/or activity in the amygdala in rats^16^. Additionally, HDAC inhibition in the basolateral amygdala decreased fear extinction and increased memory consolidation in rodents^17^. However, to the best of our knowledge, HDAC expression has not been examined in the amygdala in humans.

The amygdala represents an important neural substrate in BD due to its role in mood and emotion regulation^18–21^. Extant neuroimaging studies demonstrate structural and functional abnormalities of the amygdala in BD^22–24^, as well as within broader fronto-limbic neural circuitry^18,19^, critical for top-down regulation of emotion and attentional functions. Notably, emotion dysregulation and attention disturbance are present in BD during both acute manic and depressive episodes but also persist during periods of relative euthymia^25,26^. Given that current medications do not adequately improve these processes for many patients with BD^27^, molecular strategies to improve emotion regulation and attention are acutely needed. The implication of HDACs in emotion and attention has been shown in both rodents^12,28–35^ and humans^36,37^, however, the relationship between HDAC expression and these clinical features has not been examined in patients with BD.

To fill the translational gap that exists between genetic, pharmacological and pathological studies demonstrating roles for HDACs in BD, we measured in vivo HDAC distribution and relative expression levels in BD compared to healthy age- and sex-matched controls (CON) using the HDAC-specific radiotracer [^11^C]Martinostat^38–41^ and positron emission tomography (PET). Our primary hypothesis was that HDACs would be differentially expressed in the amygdala of BD compared to CON with potential right lateralization, based on previous functional imaging findings of amygdalae activation^42^ and right hemisphere disturbances often reported in BD^43^. In a previous study, relative in vivo HDAC expression was shown to be lower in the DLPFC in patients with schizophrenia compared to unaffected controls^36^. This finding aligned with results from a separate large-scale postmortem study^14^, which also found no differences in this region in BD. Thus, we further hypothesized that relative in vivo HDAC expression in the DLPFC would not be different between BD compared to CON. We also conducted voxelwise analyses to assess HDAC expression across the entire brain in an exploratory follow-up. In addition, to understand the clinical implications of HDACs in BD, we investigated the relationships between HDAC expression, emotion dysregulation, and attention disturbance, as measured by the Measurement and Treatment Research to Improve Cognition in Schizophrenia (MATRICS) consensus cognitive battery (MCCB)^44–47^.

## Methods

### Study design

The main goal of this study was to measure in vivo HDAC expression using [^11^C]Martinostat and simultaneous MR-PET neuroimaging. This study was approved by the Partners HealthCare Institutional Review Board (IRB) and the Massachusetts General Hospital (MGH) Radioactive Drug Research Committee. All participants provided written informed consent according to the Declaration of Helsinki. Participants underwent a physical examination by a licensed physician or nurse practitioner in order to determine study eligibility and to record medical history, medication use and smoking status. Eleven participants with BD and 11 age- and sex-matched CON (Table 1) completed a [^11^C]Martinostat MR-PET scan at the Athinoula A. Martinos Center for Biomedical Imaging. Imaging studies were not blinded to diagnosis, and no outliers were excluded (as assessed via the ROUT method^48^ in GraphPad Prism version 8; the ROUT method combines robust nonlinear regression and outlier detection based on false discovery rate to determine outliers).

**Table 1:** Demographic characteristics, medication, administered radiotracer dose, and cognitive metrics of study participants.

Demographic or cognitive metric	Bipolar	Control	*p*-value
Age (year)	38.2 ± 15.5	38.4 ± 15.3	0.91
Sex (M/F)	4/7	4/7	>0.999
Body mass index	31.4 ± 8.9	26.0 ± 4.3	0.24
Smoking status (%)	0	0	–
Parental socioeconomic index^a^	2.3 ± 0.8	3.1 ± 1.1	0.06
Handedness (L/R)^b^	1/10	0/9	>0.999
Lithium/lamotrigine (%)	55	0	–
Antipsychotics (%)	73	0	–
Chlorpromazine equivalent dose (mg/d)	193.9 ± 352.7	–	–
Injected dose (mCi)	5.3 ± 0.2	5.0 ± 0.3	0.0008
Injected mass (μg)	1.6 ± 0.9	1.1 ± 0.5	0.22
Molar activity (mCi/nmol)	1.8 ± 1.4	1.9 ± 0.9	0.37
MCCB speed of processing T-score	50.8 ± 16.9	52.4 ± 11.3	0.75
MCCB attention/vigilance T-score	46.8 ± 9.3	46.4 ± 11.8	0.93
MCCB working memory T-score	45.5 ± 14.0	47.6 ± 14.1	0.75
MCCB verbal learning T-score	51.2 ± 9.5	49.4 ± 6.7	0.40
MCCB visual learning T-score	52.8 ± 14.8	58.3 ± 10.4	0.32
MCCB reasoning and problem solving T-score	49.4 ± 10.6	45.9 ± 8.3	0.47
MCCB emotion regulation T-score	48.8 ± 6.7	45.9 ± 13.6	0.72
MCCB overall composite T-score	48.9 ± 13.9	49 ± 13.5	0.87

*p* values were determined by the Wilcoxon rank-sum test for all except sex and handedness for which a Fisher’s exact test was used. Values are reported as mean ± standard deviation unless otherwise stated.

*MCCB* measurement and treatment research to improve cognition in schizophrenia (MATRICS) consensus cognitive battery.

^a^The Hollingshead Four-Factor Index of Social Status^65^ was used to measure parental socioeconomic status (not available for 2 CON).

^b^Handedness was not available for 2 CON.

### Study participants

BD and CON were group matched for age and sex. In order to obtain group matched CON, the CON were pooled from two studies. Participants were physically healthy as determined by medical history and a physical examination. Patients with BD met DSM-IV criteria for BD 1 (*n* = 6) or BD 2 (*n* = 5). BD diagnosis was confirmed by the Structured Clinical Interview for DSM-IV-TR Axis I Disorders, Research Version, Patient Edition (SCID-I/P) or clinician confirmation. CON had no history of major physical or neuropsychiatric illness (CON were from two studies that used different methods to determine history of neuropsychiatric illness: *n* = 9 determined by SCID-I, Non-Patient Edition (SCID-I/NP) and *n* = 2 by the Mini-International Neuropsychiatric Interview (MINI)). No participants included in this study were currently taking valproic acid, a well-known HDAC inhibitor. All psychotropic medications or hormone treatments were exclusionary for CON participants. Antipsychotic mediations were permitted in BD and chlorpromazine (CPZ) equivalent doses^49^ were calculated. Six of 11 BD were taking lithium or lamotrigine, which have demonstrated potential inhibitory effects on HDACs in vitro^10,11^ (Table 1). BD medication usage is reported in Supplementary Table 1. Eligible participants were not using illicit drugs or recreational marijuana, confirmed by a urine drug screen on the day of the scan (Discover Plus Drug Test Card DIS-DOA-3124, American Screening Corp.). Furthermore, all participants met requirements for both MR and PET scanning safety regulations. Eligible female participants had a negative serum pregnancy test (Sure-Vue serum hCG-STAT, Fisher HealthCare) on the day of the scan.

### Radiosynthesis of [^11^C]Martinostat

[^11^C]Martinostat was synthesized through reductive amination, followed by conversion into a hydroxamic acid in the presence of hydroxylamine and sodium hydroxide in accordance with cGMP guidelines as described in^39^. [^11^C]Martinostat is a hydroxamic acid-based HDAC inhibitor containing an adamantyl group and radiolabeled with ^11^C.

### MR-PET data acquisition

[^11^C]Martinostat was injected through an intravenous catheter in the antecubital vein by a licensed nuclear medicine technologist. PET and MR images were acquired simultaneously on a 3T Siemens TIM Trio with a BrainPET insert. PET data were collected for 90 min post-injection. The intrinsic spatial resolution of PET in the center field-of-view was <3 mm^50^. For the MR data, an 8-channel head coil was used. A high-resolution T1-weighted anatomical scan, multi-echo magnetization prepared rapid acquisition gradient echo (MEMPRAGE) with prospective motion correction (using EPI-based volumetric navigators, vNavs), with TR = 2530 ms, TE[1-4] = 1.66 ms, 3.53 ms, 5.4 ms, 7.27 ms, FOV = 280 mm, flip angle = 7 deg, voxel size = 1 mm isotropic^51^ was acquired.

### Cognitive assessment

Participants completed the MCCB^44,45^. The Mayer-Salovey-Caruso Emotional Intelligence Test (MSCEIT) ^52^: Managing Emotions subtest was used to assess emotion dysregulation (as in refs. ^36,37^). The Continuous Performance Test – Identical Pairs^53^ was used to assess sustained attention, which is often affected in BD^54^. MCCB T-scores were age- and sex-corrected. All participants with BD and 9 CON completed the MCCB within 1 month before or after the scan. Two CON were from a study that did not include the MCCB. One of the two CON was re-contacted and completed the MSCEIT within 2 years of the scan. The MSCEIT score has been reported to remain stable over a 5-year period^55^, therefore we expect the results to be stable with a 2-year time period between cognitive and PET data collection for this CON.

### MR data processing and analysis

The MEMPRAGE images were reconstructed using FreeSurfer’s automated segmentation and parcellation (version 6.0; http://surfer.nmr.mgh.harvard.edu/). The regions of interest (ROI) for the amygdala in native space were defined using these segmentations^56^ and visually inspected. Volumes were corrected for estimated total intracranial volume (eTIV) (corrected as a ratio of volume/eTIV). Because previous morphometry MR imaging studies detected reduced volume in multiple regions of the frontal cortex as well as the amygdala, hippocampus, and thalamus in BD compared to CON^57,58^, we investigated the relationship between volume and [^11^C]Martinostat uptake. Volumes of anatomical regions represented in posthoc regions showing differences in [^11^C]Martinostat uptake between groups, or in correlations with [^11^C]Martinostat uptake, were extracted using Freesurfer tools.

### PET data processing and analysis

PET images were reconstructed using the Ordinary Poisson Ordered Subset Expectation Maximization 3D algorithm from prompt coincidences, corrected for normalization, dead time, isotope decay, photon attenuation, and expected random and scatter coincidences. MR-based attenuation correction was applied using Statistical Parametric Mapping (SPM)–based, pseudo–computed tomography^59^. PET data were binned and reconstructed in units of SUV in 1.25 mm isotropic voxel size^60^. SUV maps normalized by the whole-brain mean^37^ (excluding cerebrospinal fluid) (SUVR) were generated from 60 to 90 min post radiotracer injection. There was no difference in mean whole-brain SUV between the two groups (BD mean ± standard deviation: 3.44 ± 0.73; CON mean ± standard deviation: 3.80 ± 0.73; *U* = 44, *p* = 0.30). Motion was assessed by calculating the absolute frame displacement between six 5-min frames of the PET window of interest (i.e. 60–90 min post radiotracer injection) and a reference frame. Motion estimates were not different between groups (BD mean ± standard deviation: 0.86 ± 0.45 mm; CON mean ± standard deviation: 0.79 ± 0.43 mm; *U* = 60, *p* > 0.999). The amount of motion is below the intrinsic spatial resolution of PET.

ROI analysis was used to quantify differences in [^11^C]Martinostat uptake between BD and CON. The left and right amygdala were selected as a priori ROIs based on evidence of structural and functional abnormalities in the amygdala in BD^23,24,61^ and lateralized amygdala functions^42^. In order to account for the small size of the amygdala and possible PET signal spillover from neighboring tissue, we applied geometric transfer matrix (GTM), a region-based partial volume correction (PVC) method using PETSurfer tools available within FreeSurfer^62,63^. The PVC SUVR values were extracted from the left and right amygdala in native space. The DLPFC was also selected as an a priori ROI based on previous findings in postmortem brain tissue of differences in *HDAC2* mRNA expression in donors with schizophrenia but not donors with BD compared to controls^14^. Individual SUVR maps were registered to MNI standard space and spatially smoothed at full width at half maximum (FWHM) 8 mm. The DLPFC SUVR values were extracted from MNI standard space with the same DLPFC ROI used previously in Gilbert et al.^36^.

Furthermore, to comprehensively interrogate in vivo HDAC expression in BD, we also conducted exploratory voxelwise analyses (described below in Statistical analysis).

### Statistical analysis

Wilcoxon rank-sum test was used to assess between-group differences in demographic scores, MCCB T-scores, [^11^C]Martinostat uptake (SUV/SUVR), and volumetric data. Between-group differences in the ROI analysis were assessed with residuals of SUVR values (in the amygdala and DLPFC) after controlling for age and sex using Matlab’s fitlm linear regression function.

A whole-brain voxelwise group comparison for [^11^C]Martinostat uptake between BD and CON was conducted using FSL’s FEAT (FMRIB software library, Oxford, UK; https://fsl.fmrib.ox.ac.uk/fsl/) with an unpaired *t* test, ordinary least squares (OLS) mixed-effects modeling, a significance threshold of *Z* > 2.3, and cluster correction of *p*_cluster_ < 0.05^64^. Age and sex were added to the model as regressors of no interest. Whole-brain voxelwise analyses correlating [^11^C]Martinostat uptake with MCCB emotion regulation and attention T-scores in BD was conducted using FSL’s FEAT (*Z* > 2.3, *p*_cluster_ < 0.05), with age and sex added to the model as regressors of no interest.

Spearman’s rank-order correlation was used to correlate: (1) right amygdalar SUVR residuals with CPZ equivalent dose in BD, (2) left and right amygdalar SUVR residuals with MCCB emotion regulation and attention T-scores in BD and across the whole sample (BD and CON), and (3) SUVR residuals, MCCB emotion regulation and attention T-scores with volumes of anatomical regions represented in posthoc regions.

All statistical tests other than whole-brain voxelwise analyses were performed using GraphPad Prism version 8.

### Exclusion of controls in whole-brain voxelwise correlation analyses

No correlations between [^11^C]Martinostat uptake and MCCB emotion regulation T-scores and MCCB attention T-scores were found across the whole sample (BD and CON), or between [^11^C]Martinostat uptake and MCCB attention T-scores in CON in whole-brain voxelwise analyses using FSL’s FEAT (*Z* > 2.3, *p*_cluster_ < 0.05), with age and sex added to the model as regressors of no interest. The association between [^11^C]Martinostat uptake and MCCB emotion regulation in CON was previously reported in^37^.

## Results

### Demographics and clinical characteristics

Demographic information and cognitive metrics of participants are provided in Table 1 (*n* = 11 participants per group). No differences in age, sex, body mass index (BMI), smoking status, parental socioeconomic status^65^, or cognitive performance as assessed by the MCCB were detected between groups. Information on parental socioeconomic status and handedness was not available for 2 CON because they were from a study that did not inquire this information. The injected [^11^C]Martinostat dose was significantly different between BD and CON. As injected dose is included in the calculation of SUV, and whole-brain normalization which removes inter-individual differences in global signal was used to calculate SUVR, a 6% difference in injected dose across the two groups will not impact between-group differences. Medication usage of participants with BD is detailed in Supplementary Table 1.

### Region of interest analyses

**Fig. 1:**
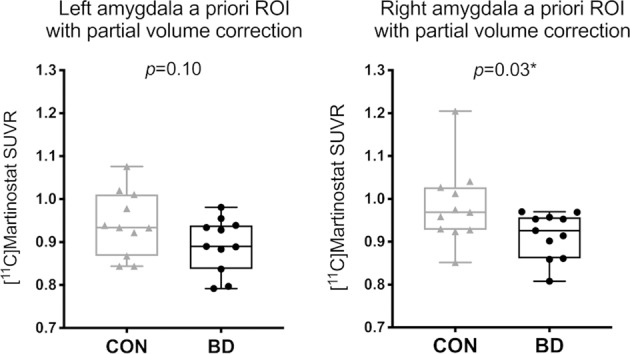
[^11^C]Martinostat uptake is lower in the right amygdala of participants with bipolar disorder (BD) compared to matched healthy controls (CON). SUVR extracted from the left and right amygdala a priori ROIs in native space of BD compared to CON (*n* = 11 subjects per group). Box plots display median, first quartile, third quartile, and range of min-max. Geometric transfer matrix (GTM) partial volume correction was applied.

To characterize HDAC expression patterns between BD and CON, simultaneous MR-PET was performed with [^11^C]Martinostat, a radiotracer selective for HDAC paralogs 1, 2, 3, and putatively 6^39,41^. [^11^C]Martinostat uptake was measured using SUVR from 60 to 90 min post radiotracer injection. SUVR was lower in the right amygdala of BD compared to CON with a 7.2% mean percentage difference (*U* = 27, *p* = 0.03, Fig. 1). The difference in SUVR in the left amygdala was not statistically significant between groups (*U* = 35, *p* = 0.10). SUVR in the left and right amygdala did not correlate with motion estimates in BD and CON (left amygdala: Spearman’s *r* = 0.25, *p* = 0.26; right amygdala: Spearman’s *r* = 0.04, *p* = 0.86). There were no subjects identified as outliers using the ROUT method in GraphPad Prism. The volumes of the left and right amygdala in native space were not significantly different between groups (left: *U* = 47, *p* = 0.40; right: *U* = 49, *p* = 0.48). SUVR in the DLPFC was not significantly different between groups (*U* = 41, *p* = 0.22, Supplementary Fig. 1) and did not correlate with motion estimates in BD and CON (Spearman’s *r* = −0.10, *p* = 0.67).

#### Analyses of potential effects of medication

SUVR in the right amygdala of participants with BD was not related to lithium or lamotrigine prescription (*U* = 9, *p* = 0.33, Supplementary Fig. 2a). SUVR in the right amygdala of participants with BD was not related to CPZ equivalent dose (Spearman’s *r* = −0.06, *p* = 0.85). SUVR in the right amygdala did not differ between participants with BD 1 or BD 2 (*U* = 13, *p* = 0.79, Supplementary Fig. 2b).

### Exploratory voxelwise analysis

**Fig. 2:**
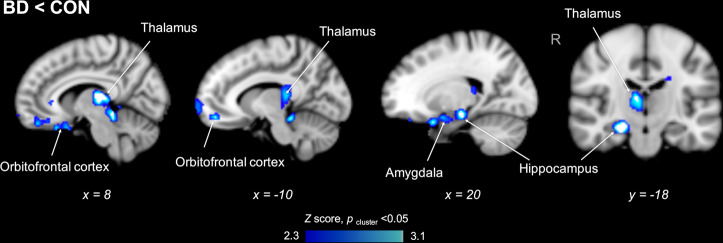
[^11^C]Martinostat uptake is lower in the bilateral thalamus, orbitofrontal cortex, right hippocampus, and right amygdala of participants with bipolar disorder (BD) compared to matched healthy controls (CON). Statistical maps from voxelwise comparison of SUVR between groups, controlled for age and sex, overlaid onto the MNI 1 mm template in radiological orientation (*Z* > 2.3, *p*_cluster_ < 0.05). Blue-light blue represents regions significantly lower in BD compared to CON (*n* = 11 subjects per group).

In a whole-brain voxelwise comparison of SUVR between groups (*Z* > 2.3, *p*_cluster_ < 0.05), participants with BD showed lower regional uptake in the bilateral thalamus, orbitofrontal cortex, right hippocampus, and right amygdala compared to CON (Fig. 2). No area showed higher SUVR in BD compared to CON. The volumes of these regions were not different between groups in native space (*U* = 43, *p* = 0.27). No between-group differences were found in the volumes of total gray matter (*U* = 53, *p* = 0.65), white matter (*U* = 40, *p* = 0.19) or cerebrospinal fluid (*U* = 48, *p* = 0.44).

### Correlations between cognition and [^11^C]Martinostat uptake

#### Emotion regulation

There was no group difference in MCCB emotion regulation T-scores between BD and CON (Table 1). There was no association between SUVR in the left or right amygdala a priori ROIs and emotion regulation in BD (left: Spearman’s *r* = 0.03, *p* = 0.93, right: Spearman’s *r* = 0.25, *p* = 0.46). In an exploratory whole-brain voxelwise analysis, higher SUVR in the right prefrontal white matter and left perisylvian region were associated with higher emotion regulation scores in BD (*Z* > 2.3, *p*_cluster_ < 0.05; Supplementary Fig. 3); the volumes of these regions were not correlated to SUVR in native space. Additionally, lower SUVR in the right middle frontal gyrus was associated with higher emotion regulation scores in BD, however, smaller volume was associated with lower SUVR (Spearman’s *r* = 0.67, *p* = 0.03) and higher emotion regulation (Spearman’s *r* = −0.70, *p* = 0.02) in native space.

#### Attention

There was no group difference in MCCB attention T-scores between BD and CON (Table 1). There was no association between SUVR in the left or right amygdala a priori ROIs and attention in BD (left: Spearman’s *r* = −0.46, *p* = 0.16, right: Spearman’s *r* = 0.16, *p* = 0.63). In an exploratory whole-brain voxelwise analysis, higher SUVR in the bilateral hippocampus and pons, right parahippocampal gyrus, left pallidum and inferior longitudinal fasciculus (temporal regions) were associated with higher attention scores; and lower SUVR in the left middle frontal gyrus, pre- and postcentral gyrus, inferior parietal lobule, and lateral occipital cortex (fronto-parietal regions) were associated with higher attention scores in BD (*Z* > 2.3, *p*_cluster_ < 0.05; Supplementary Fig. 4). Posthoc assessments of volume showed no correlations between attention and volume (temporal regions: Spearman’s *r* = 0.35, *p* = 0.30; fronto-parietal regions: Spearman’s *r* = −0.54, *p* = 0.09), or between SUVR and volume (temporal regions: Spearman’s *r* = 0.50, *p* = 0.13; fronto-parietal regions: Spearman’s *r* = 0.38, *p* = 0.25).

## Discussion

BD is characterized by recurrent episodes of altered mood involving disruptions in emotion regulation and cognitive processes which lead to overall functional impairment. A number of structural, functional, and molecular neuroimaging studies implicate aberrant fronto-limbic neural circuitry in BD^24,66–71^, however, the molecular mechanisms underlying structural and functional alterations are not fully understood. In this study we used [^11^C]Martinostat PET to measure and compare relative HDAC expression levels in BD and CON because epigenetic mechanisms, such as those regulated by HDACs, have the potential to reconcile contributions of both genetic and environmental factors in neuropsychiatric disorders including BD. Our primary results indicate lower relative HDAC expression in the right amygdala of BD, as well as within a broader fronto-limbic distribution including the thalamus, orbitofrontal cortex, and hippocampus. Moreover, relative HDAC expression was related to attention and emotion regulation selectively in BD. These results suggest a potential role for HDACs in the fundamental pathophysiology of BD as well as in a subset of its hallmark clinical features.

Consistent with our hypothesis, [^11^C]Martinostat SUVR was lower in the right amygdala of BD compared to CON. Comparatively, relative HDAC expression was not found to differ in the amygdala of individuals with schizophrenia (SCZ) or schizoaffective disorder (SAD) compared to CON in a prior PET study^36^. Additionally, relative HDAC expression was lower in the DLPFC, a brain region relevant to the pathophysiology of SCZ in a previous study using [^11^C]Martinostat in SCZ/SAD^36^, but not in the current study of BD, results that further align with postmortem data^14^. These observations suggest that lower relative HDAC expression in the right amygdala may be a specific etiological feature of BD. The group difference in SUVR of the left amygdala approached but did not reach significance and may be consistent with the hemispheric asymmetry hypothesis of BD, which implicates altered right hemisphere brain function in bipolar depression^72^. However, this result could reflect a limit in statistical power to detect medium effect sizes in the current sample.

Exploratory whole-brain voxelwise analyses detected lower relative HDAC expression in BD compared to CON including the right amygdala and hippocampus, bilateral thalamus, and orbitofrontal cortex. The amygdala, hippocampus, thalamus, and orbitofrontal cortex are involved in mood regulation, sensory integration, and decision making, which are frequent clinical presentations in patients with BD^73^. In particular, our finding in the thalamus is primarily in the mediodorsal thalamus, which is involved in cognitive processes and attention^74,75^, likely due to its dense connections to the prefrontal cortex^76^. The mediodorsal thalamus has been found to be underconnected to the prefrontal cortex in both patients with BD and schizophrenia using resting state functional MR imaging^77^. Moreover, fronto-limbic regions were previously shown to be abnormal in BD via structural, functional, and molecular neuroimaging studies as mentioned above, providing support to the hypothesis that altered HDAC expression may contribute to the observed regional abnormalities. To date, concordance between HDAC function in BD and the neural circuitry underlying behavior has only been indirectly extrapolated across different postmortem and rodent studies^15,78^. Use of [^11^C]Martinostat PET begins to fill this gap by identifying altered HDAC levels in participants with BD in vivo and also offers the unique advantage of possible application during simultaneous functional MR imaging, which should be undertaken in future studies to further dissect the potential impact of altered HDAC levels on neural circuitry in BD. Furthermore, it would be interesting to consider anatomical subregions moving forward. For instance, HDAC inhibition in the basolateral amygdala enhances memory consolidation in rats^17^. The investigation of amygdalar subnuclei may provide more insight into the molecular mechanism of HDAC regulation in relation to cognitive function in BD.

HDACs regulate genes important for activity-dependent regulation of neuroplasticity^79^, as well as genes associated with BD^80–83^. Therefore, it is possible that altered HDAC expression may contribute to emotional or cognitive disturbances characteristic of BD through altered neuroplasticity. In this study, we explored whether relative HDAC expression levels correlate with emotion regulation and attention. Whole-brain analyses revealed that SUVR was associated with MCCB emotion regulation performance, selectively in BD and within brain regions that have relevance to emotion regulation. Specifically, the left perisylvian region is implicated in language comprehension^84^, the superior temporal sulcus in multiple social processes, including theory of mind^85^, and the middle frontal gyrus in social judgment^86^. These correlates of emotion regulation in BD differ from those identified in healthy participants (e.g. the inferior longitudinal and fronto-occipital fasciculus and hippocampus^37^), raising the possibility that differential regional patterns of HDAC expression may underlie emotion dysregulation in BD. Additionally, lower SUVR in fronto-parietal regions and higher SUVR in temporal regions were correlated with higher attention scores in BD. Sustained attention is typically supported by engagement of fronto-parietal circuitry and deactivation of temporo-limbic regions, including the parahippocampal gyrus^87^. Given that a delicate balance of these two cognitive processes is likely needed for sustained attention, altered relative HDAC expression in these brain regions in BD may contribute to differences in sustained attention. Overall, our results suggest that altered relative HDAC expression in BD may have impacts on emotion regulation and attention.

We acknowledge several limitations of this work. Our study measures differences in [^11^C]Martinostat uptake relative to the whole-brain mean (SUVR) and not absolute uptake values. Therefore, future [^11^C]Martinostat PET studies with arterial blood sampling in larger sample sizes will be necessary to validate HDAC expression differences in BD. Given substantial cognitive heterogeneity in BD, the modest sample size of this study may explain why we did not see differences in cognitive performance at the group level. Another possibility is that the participants with BD in this study are more high-functioning to be able to complete a 90-min PET scan, therefore these results may not be generalizable to all patients with BD. Furthermore, participants with BD were medicated and we do not have standardized rating scales of their mood symptoms at the time of the scan. Therefore, future studies with more narrowly defined cohorts of participants with BD, including first-episode patients with limited medication exposure are needed to assess the potential impact of medication status on HDAC expression levels. Despite these limitations, we did test potential confounding factors such as anatomical volumes and potential effects of medication, and our findings were robust to these issues. Nonetheless, a larger sample size will be needed to confirm our findings.

In conclusion, our study presents the first in vivo evidence of altered relative HDAC expression in fronto-limbic regions between participants with BD and age- and sex-matched healthy CON. This work suggests a potential link between altered HDAC expression, attention, and emotion dysregulation in BD.

## Supplementary information

Supplementary figure legends

Supplementary Table

Figure S1

Figure S2

Figure S3

Figure S4

## Data Availability

The data that support these findings are available from the corresponding author, J.M.H., upon reasonable request. Human subject data will be deidentified to protect confidentiality.

## Code availability

Custom codes for PET and MR data processing are available from the corresponding author, J.M.H., upon reasonable request.
