# Supplementary material for: In vivo human brain expression of histone deacetylases in bipolar disorder
Source: Transl Psychiatry. 2020 Jul 8;10:224. doi: 10.1038/s41398-020-00911-5 (PMC7343804; doi:10.1038/s41398-020-00911-5)
Supplement: Supplementary file 1 — Supplementary figure legends [file 41398_2020_911_MOESM1_ESM.docx]

**Supplementary figure legends**

**Supplementary Fig. 1.** [^11^C]Martinostat uptake in the dorsal lateral prefrontal cortex is not different between participants with bipolar disorder (BD) compared to matched healthy controls (CON). SUVR extracted from the dorsal lateral prefrontal cortex of BD compared to CON (n=11 subjects per group). Box plots display median, first quartile, third quartile, range of min-max.

**Supplementary Fig. 2.** [^11^C]Martinostat uptake in the right amygdala of participants with BD is not associated with lithium/lamotrigine prescription or BD diagnosis type. **a** SUVR in the right amygdala of BD participants with lithium/lamotrigine prescription are shown in open circles. **b** SUVR in the right amygdala of participants with BD 1 are indicated in black circles and BD 2 are indicated in red circles.

**Supplementary Fig. 3.** [^11^C]Martinostat uptake is associated with emotion regulation assessed by the Measurement and Treatment Research to Improve Cognition in Schizophrenia (MATRICS) consensus cognitive battery (MCCB) in participants with BD. Voxelwise correlations of SUVR with MCCB emotion regulation T-scores in participants with BD, controlled for age and sex (n=11). Statistical maps were overlaid onto the MNI 1mm template in radiological orientation (*Z*>2.3, *p_cluster_*<0.05). Red-yellow represents regions where SUVR significantly increased with MCCB emotion regulation T-scores and blue-light blue represents regions where SUVR significantly decreased with MCCB emotion regulation T-scores.

**Supplementary Fig. 4.** [^11^C]Martinostat uptake is associated with attention assessed by the Measurement and Treatment Research to Improve Cognition in Schizophrenia (MATRICS) consensus cognitive battery (MCCB) in participants with BD. Voxelwise correlations of SUVR with MCCB attention T-scores in participants with BD, controlled for age and sex (n=11). Statistical maps were overlaid onto the MNI 1mm template in radiological orientation (*Z*>2.3, *p_cluster_*<0.05). Red-yellow represents regions where SUVR significantly increased with MCCB attention T-scores and blue-light blue represents regions where SUVR significantly decreased with MCCB attention T-scores.
