# Supplementary material for: In vivo human brain expression of histone deacetylases in bipolar disorder
Source: Transl Psychiatry. 2020 Jul 8;10:224. doi: 10.1038/s41398-020-00911-5 (PMC7343804; doi:10.1038/s41398-020-00911-5)
Supplement: Supplementary file 2 — Supplementary Table [file 41398_2020_911_MOESM2_ESM.docx]

**Supplementary Table 1.** Prescribed medication of participants with bipolar disorder (BD).

| **Clinical Characteristic** | **Participants with Bipolar Disorder (n=11)** |
| --- | --- |
| **Psychiatric Diagnoses (BD 1: BD 2)** | 6:5 |
| **Second-Generation Antipsychotics** | Aripiprazole (3) |
|  | Quetiapine (3) |
|  | Lurasidone (1) |
|  | Risperidone (1) |
| **First-Generation Antipsychotics** | NA |
| **Antidepressants** | Bupropion (4) |
|  | Duloxetine (1) |
|  | Escitalopram (1) |
|  | Fluvoxamine (2) |
|  | Mirtazapine (1) |
|  | Sertraline (1) |
|  | Trazodone (1) |
| **Anticonvulsants, Anxiolytics, Sedatives** | Lamotrigine (5) |
|  | Alprazolam (1) |
|  | Buspirone (1) |
|  | Carbamazepine (1) |
|  | Clonidine (2) |
|  | Diazepam (1) |
|  | Lorazepam (1) |
|  | Oxazepam (1) |
|  | Oxcarbazepine (1) |
|  | Topiramate (1) |
|  | Clonazepam (1) |
|  | Zolpidem Tartrate (1) |
| **Stimulants** | Methylphenidate (1) |
|  | Amphetamine/Dextroamphetamine (1) |
|  | Lisdexamfetamine (1) |
| **Mood Stabilizers** | Lithium Carbonate (2) |
