# Supplementary figures and images for: In vivo human brain expression of histone deacetylases in bipolar disorder
Source: Transl Psychiatry. 2020 Jul 8;10:224. doi: 10.1038/s41398-020-00911-5 (PMC7343804; doi:10.1038/s41398-020-00911-5)

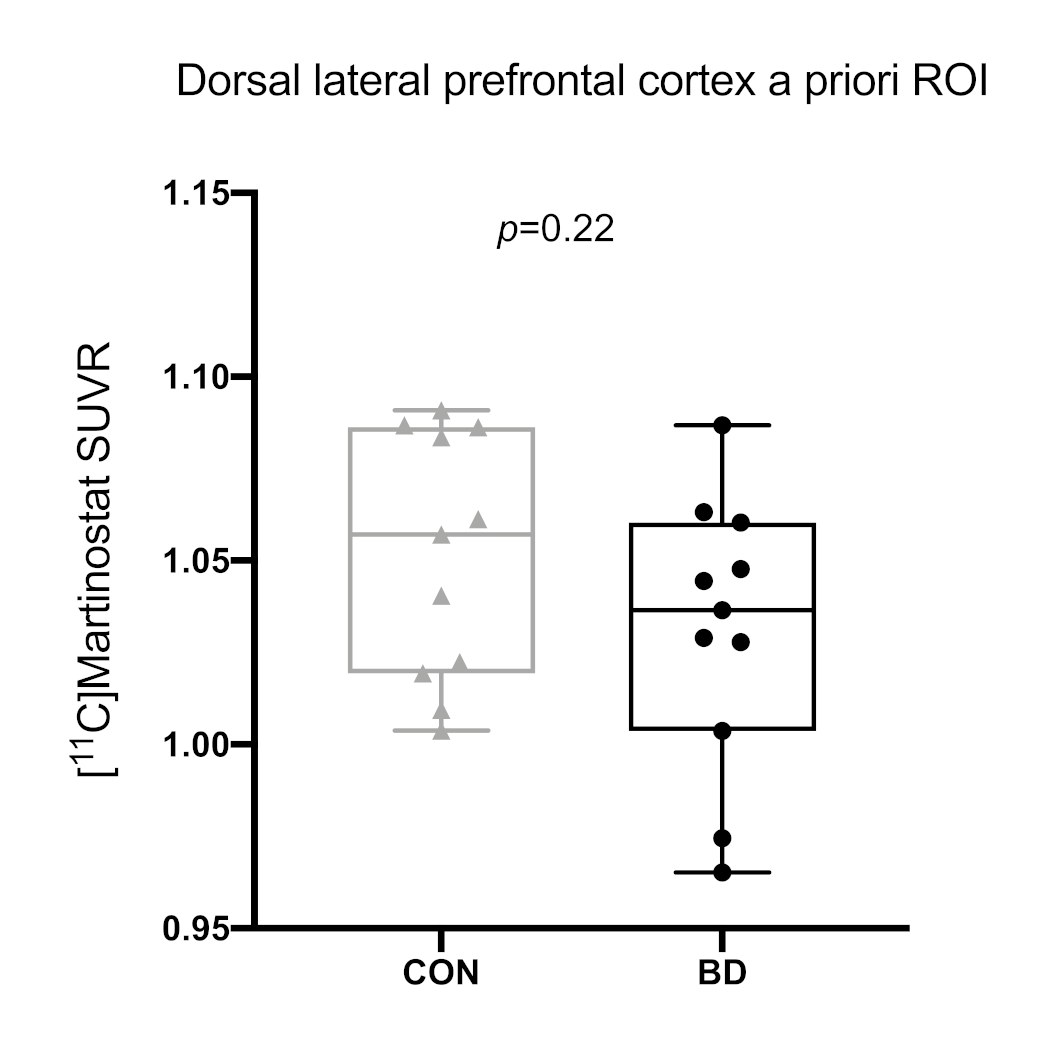

Supplement: Supplementary file 3 — Figure S1 [file 41398_2020_911_MOESM3_ESM.tif]

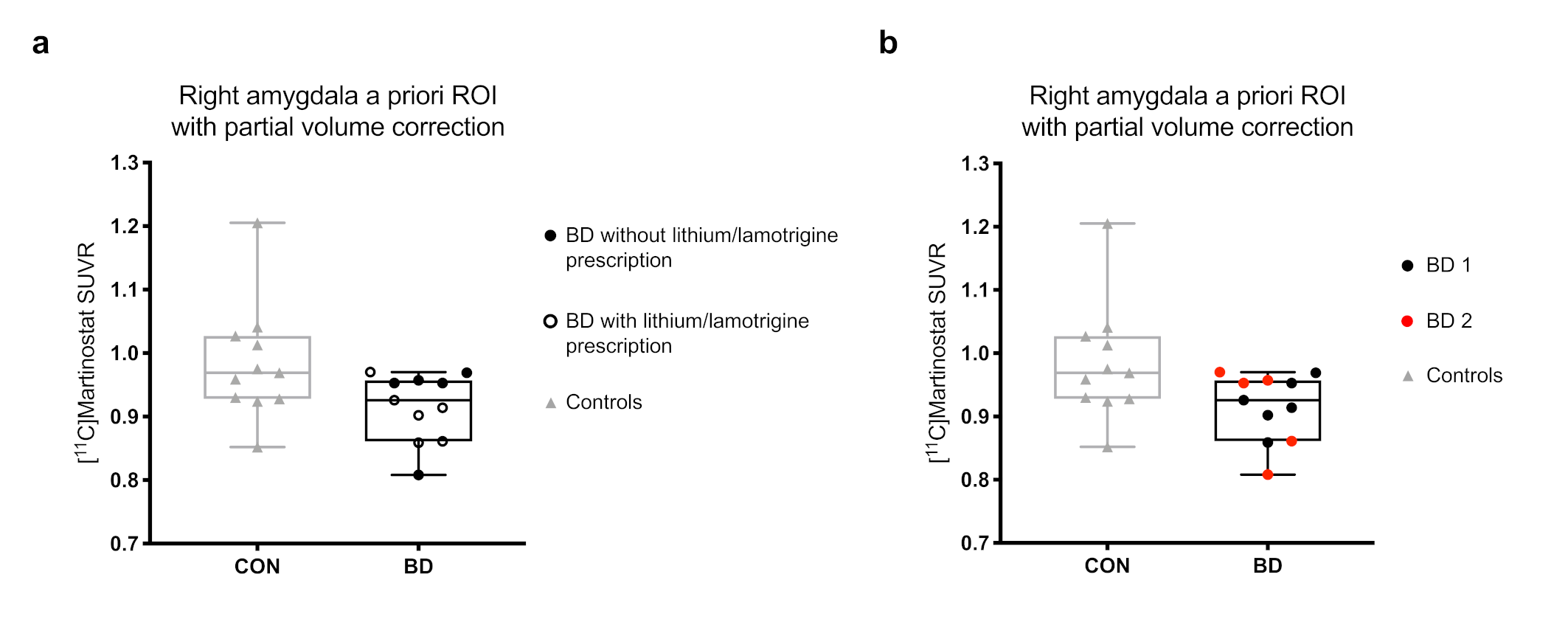

Supplement: Supplementary file 4 — Figure S2 [file 41398_2020_911_MOESM4_ESM.tif]

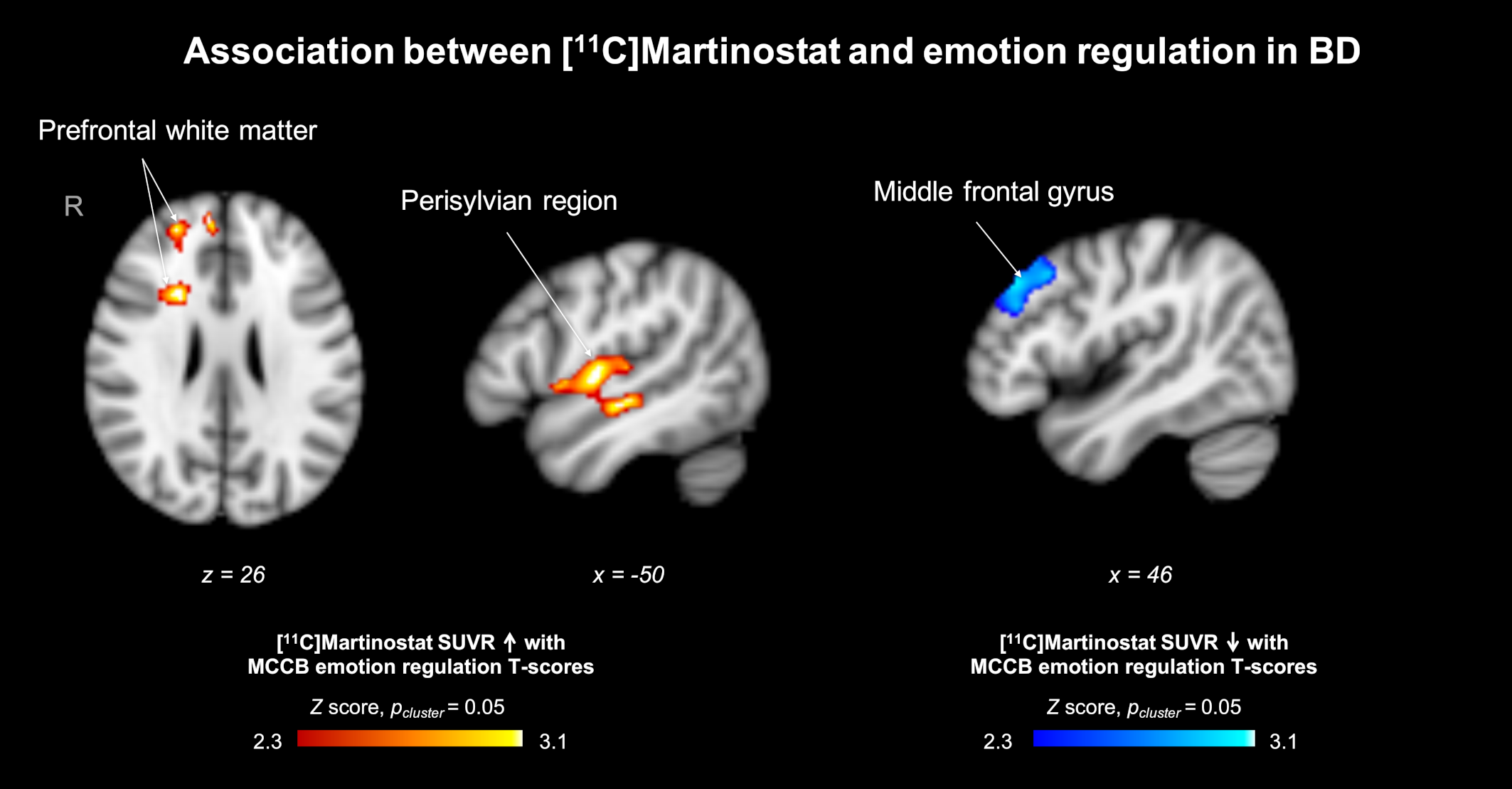

Supplement: Supplementary file 5 — Figure S3 [file 41398_2020_911_MOESM5_ESM.tif]

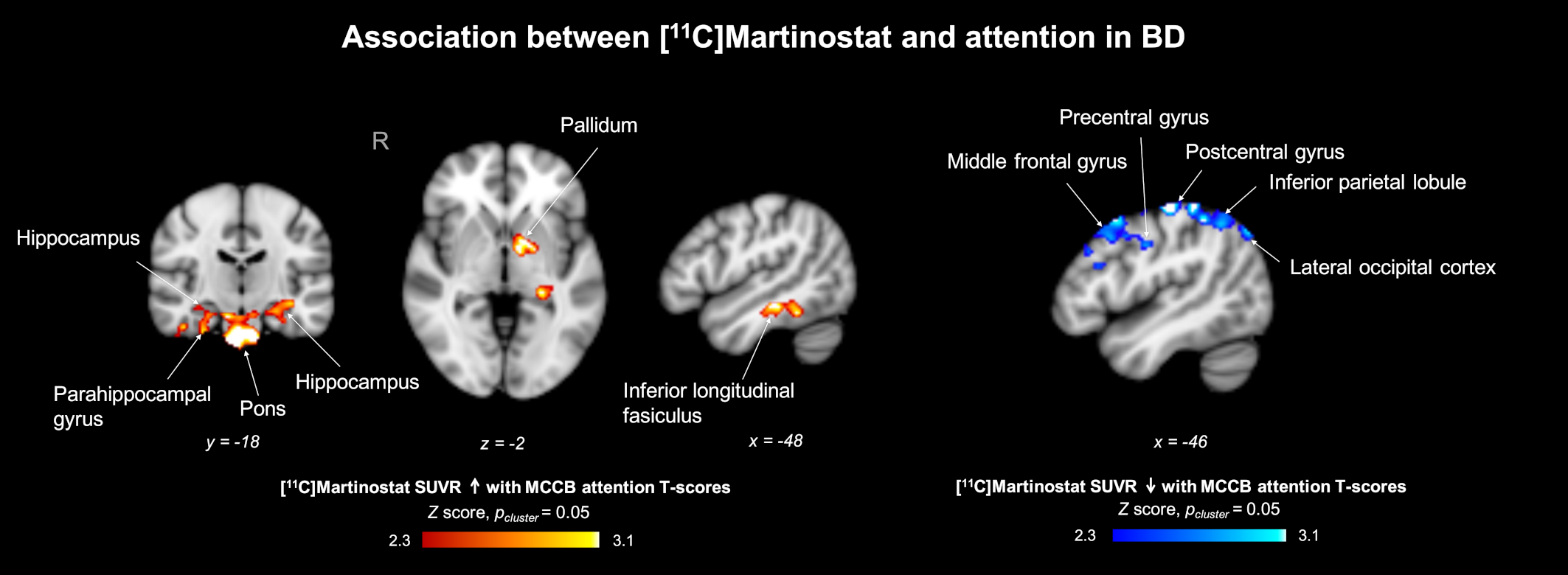

Supplement: Supplementary file 6 — Figure S4 [file 41398_2020_911_MOESM6_ESM.tif]
